# Descriptive Analysis of LAP1 Distribution and That of Associated Proteins throughout Spermatogenesis

**DOI:** 10.3390/membranes7020022

**Published:** 2017-04-07

**Authors:** Joana B. Serrano, Filipa Martins, João C. Sousa, Cátia D. Pereira, Ans M. M. van Pelt, Sandra Rebelo, Odete A. B. da Cruz e Silva

**Affiliations:** 1 Neuroscience and Signaling Laboratory, Department of Medical Sciences, Institute for Biomedicine—iBiMED, University of Aveiro, 3810-193 Aveiro, Portugal; jmbs@ua.pt (J.B.S.); samartins@ua.pt (F.M.); daniela.pereira@ua.pt (C.D.P.); odetecs@ua.pt (O.A.B.-d.C.S.); 2 Life and Health Sciences Research Institute (ICVS), School of Health Sciences, University of Minho, 4710-057 Braga, Portugal; jcsousa@med.uminho.pt; 3 ICVS/3B’s—PT Government Associate Laboratory, University of Minho, 4710-057 Braga/Guimarães, Portugal; 4 Center for Reproductive Medicine, Academic Medical Center, University of Amsterdam, 1105 AZ Amsterdam, The Netherlands; a.m.vanpelt@amc.uva.nl

**Keywords:** nuclear envelope, lamina-associated polypeptide 1, lamins, protein phosphatase 1, manchette, spermatids

## Abstract

Spermatogenesis comprises highly complex differentiation processes. Nuclear envelope (NE) proteins have been associated with these processes, including lamins, lamina-associated polypeptide (LAP) 2 and the lamin B-receptor. LAP1 is an important NE protein whose function has not been fully elucidated, but several binding partners allow predicting putative LAP1 functions. To date, LAP1 had not been associated with spermatogenesis. In this study, LAP1 expression and cellular/subcellular localization during spermatogenesis in human and mouse testes is established for the first time. The fact that LAP1 is expressed during nuclear elongation in spermiogenesis and is located at the spermatids’ centriolar pole is singularly important. LAP1 binds to members of the protein phosphatase 1 (PP1) family. Similar localization of LAP1 and PP1γ2, a testis-specific PP1 isoform, suggests a shared function for both proteins during spermiogenesis. Furthermore, this study suggests an involvement of LAP1 in manchette development and chromatin regulation possibly via interaction with acetylated α-tubulin and lamins, respectively. Taken together, the present results indicate that, by moving to the posterior pole in spermatids, LAP1 can contribute to the achievement of non-random, sperm-specific chromatin distribution, as well as modulate cellular remodeling during spermiogenesis. In addition, LAP1 seems to be associated with dynamic microtubule changes related to manchette formation and flagella development.

## 1. Introduction

Spermatogenesis is a highly complex and unique differentiation process, involving germ cell proliferation and renewal, meiosis and spermiogenesis, under the regulation of Sertoli cells in the seminiferous tubules, as well as Leydig and peritubular cells in the interstitium. During spermatogenesis, undifferentiated diploid spermatogonia undergo intricate morphological and biochemical transformations, leading to the formation of highly specialized cells, the haploid spermatozoa [1]. The morphological changes that occur during spermiogenesis are particularly interesting. This process includes four crucial phases: Golgi, capping, acrosomal and maturation (reviewed in [2]). Together, these highly heterogeneous phases enable the identification of specific stages (designated I–XII) in the spermatogenic cycle of the seminiferous epithelium. The most prominent morphologic nuclear changes in spermatids involve chromatin condensation. Firstly, chromosomes become packed, tighter and stain more intensely during the acrosomal phase. Throughout the maturation phase, the nucleus continues to condense, and the acrosome matures into a thin structure that covers nearly all the nucleus. Lastly, the cytoplasm forms lobes that are ultimately excised as residual bodies (reviewed in [2,3,4]). Based on particular cellular features found in each spermiogenic phase, which allow differentiating between round, elongating and elongated mature spermatids, it is possible to assess the spermatogenic stage displayed by a certain seminiferous tubule and identify germ cells from prior stages of the differentiation process, such as spermatogonia and spermatocytes. Of note, although the cycle of spermatogenesis in mouse testis (for more detailed information, see [2]) is easily analyzed by immunohistochemical sectioning techniques, the spermatogenic stages in human testis (for more detailed information, see [5]) are harder to identify due to their organization into more complex configurations.

The role of several nuclear envelope (NE) proteins has been investigated throughout spermatogenesis. Specifically, lamins, lamina-associated polypeptide (LAP) 2, the lamin B-receptor (LBR) and elements of the linker of nucleoskeleton and cytoskeleton (LINC) complex are amongst the NE proteins with more prominent functional associations with the spermatogenic cycle [6,7,8,9,10,11,12]. LAP1 is an integral inner nuclear membrane (INM) protein, encoded by the *TOR1AIP1* gene, that binds directly to lamins and indirectly to chromosomes [13,14]. However, despite being one of the first LAPs identified [13], the physiological functions of this ubiquitously-expressed NE protein remain poorly understood. Nevertheless, LAP1 has been implicated in the regulation of the NE structure, cell cycle progression, mitosis, NE localization of torsinA and regulation of its ATPase activity, as well as skeletal muscle maintenance [14,15,16]. In our previous work, LAP1 was found in the mitotic spindle, mid-body and centrosomes, indicating a strong functional association between LAP1 and these cell cycle-related components. Specifically, LAP1 colocalizes with γ-tubulin in centrosomes at metaphase and anaphase during mitosis. In addition, LAP1 depletion seems to be correlated with altered centrosome positioning and a decrease in the number of mitotic cells [15]. LAP1 association with cell cycle progression is particularly interesting as it shares functional elements with spermatogenesis, in particular with meiosis.

Through the study of LAP1 interacting proteins, it was possible to extrapolate biological functions inherent to LAP1 itself [17]. For instance, an important LAP1 interactor is the major eukaryotic protein phosphatase 1 (PP1) [18]. Regulation of PP1 cellular functions is determined by the formation of complexes with different PP1 interacting proteins (PIPs), and LAP1 has been identified as a novel PIP, specifically as a PP1-substrate [18,19]. In mammals, there are four PP1 isoforms: PP1α, PP1β/δ and the splice variants PP1γ1 and PP1γ2 [20]. PP1γ2 is an important testis-specific and sperm-enriched isoform associated with spermatogenesis and sperm motility [21,22]. PP1 isoform expression profiles vary in mouse testis depending on the cell type, but its presence in human spermatogenesis has never been demonstrated. In mouse, PP1γ1 and PP1α expressions are restricted to spermatogonia, pachytene spermatocytes and somatic cells. Remarkably, PP1γ2 is the only isoform present in secondary spermatocytes, round spermatids and elongating spermatids [23,24].

LAP1 also binds directly to structural protein components of the NE, the nuclear lamins [14]. The composition and properties of the nuclear lamina in spermatogenic cells differ significantly from those of somatic cells in the same species [25,26]. For instance, the presence and distribution of A-type lamins (lamins A and C) in mouse testis has been investigated, but the results are inconsistent. Overall, lamin A/C has been described in Sertoli and Leydig cells [27], pachytene spermatocytes [28] and in the perinuclear region of elongated spermatids [28,29]. Nonetheless, Shen and colleagues have shown that the decreased expression of lamin A/C caused a significant increase in sperm head abnormalities [29]. In addition, a rodent meiosis-specific A-type lamin, the isoform C2, was found to regulate chromosome dynamics and to promote efficient homologous recombination in mouse [30,31]. Conversely, B-type lamins are generally expressed during the whole mouse spermatogenic cycle [27,28], but lamin B1 in particular has never been investigated in mouse spermatogenesis. Lamin B3, a testis-specific isoform, is present in mouse spermiogenesis, concentrating at the posterior pole of elongating spermatids [32]. Nuclear lamin distribution in human spermatogenesis is still not clear. Machiels and colleagues disclosed that, while both A- and B-type lamins were present in Sertoli, Leydig and peritubular cells, only B-type lamins were expressed in spermatogonia, and A-type lamins were solely observed in spermatocytes [33]. Alternatively, Elkhatib et al. confirmed the presence of A-type lamins only in somatic cells. In addition, this group demonstrated that lamins B1, B2 and, probably, B3 are all expressed in the nuclear periphery of human spermatids, accumulating at the posterior pole during the maturation process [26]. Therefore, lamin B1 in particular was only associated with human spermiogenesis. Overall, the cellular distribution of lamins A/C and B1 in the various stages of spermatogenesis is not fully elucidated and should be clarified.

Given the association of LAP1 with cellular dynamics, such as cell cycle progression and mitosis, and the fundamental role that NE proteins play in spermatogenesis, the present work investigates the expression and cellular/subcellular localization of LAP1 in the spermatogenic cycle both in mouse and human testes. Additionally, known functionally-associated LAP1 interactors were also analyzed, namely PP1γ2, lamin A/C and lamin B1. Due to the functional association of LAP1 with mitotic spindle organization during mitosis [15,34], the distribution of γ-tubulin, a marker of the centriolar pole in spermatids [35], and also acetylated α-tubulin during spermatogenesis was likewise analyzed.

## 2. Results

### 2.1. LAP1 Expression and Localization in the Testis

**Figure 1:**
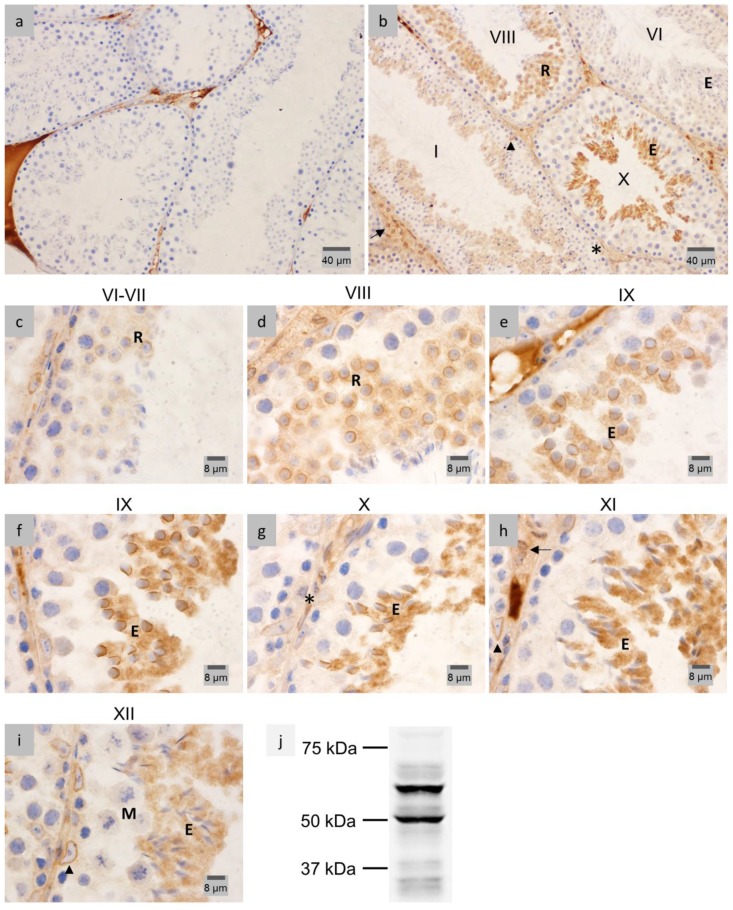
Immunohistochemical localization and expression of LAP1 in mouse testis sections fixed in diluted Bouin’s solution. Positive immunostaining appears in brown color and nucleus counterstaining with hematoxylin. (**a**) Negative controls with rabbit IgG showed LAP1 staining only in the interstitial tissue. (**b**) All epithelial stages were examined, of which stages I, VI, VIII and X (stages are indicated with Roman numerals) are depicted. LAP1 is present in the nuclear envelope (NE) of peritubular (asterisk), Leydig (arrow), and Sertoli (arrowhead) cells. LAP1 polarizes at one half of the NE in round spermatids (R) during stage VIII, then presents high positive staining in the cytoplasm of stage X elongating spermatids (E), which later show decreased cytoplasmic LAP1 staining in stage I. At the final stage of spermatogenesis, stage VI fully-elongated spermatids (E) apparently do not stain for LAP1. (**c**) During stages VI–VII, LAP1 is concentrated in the NE and less prominently in the cytoplasm of round spermatids (R). (**d**) In stage VIII, LAP1 signal polarizes at one half of the NE of round spermatids (R), with some cytoplasmic LAP1 staining being also observed. (**e**) Early and (**f**) late stage IX elongating spermatids (E) start displaying a shift in the nuclear direction towards the tubule lumen; simultaneously, LAP1 expression accentuates in one half of the NE and spreads to the cytoplasm. (**g**) In stage X, LAP1 still concentrates in one half of the NE of elongating spermatids (E), and its cytoplasmic presence is sustained. LAP1 staining is also observed in the NE of peritubular cells (asterisk). (**h**) Stage XI elongating spermatids (E) show a homogeneous distribution of LAP1 in the cytoplasm, contrasting with a decrease in the NE when compared to the previous stages. Sertoli (arrowhead) and Leydig (arrow) cells are also stained for LAP1. (**i**) In stage XII seminiferous tubules, with representative meiotic cells (M), LAP1 is located in the cytoplasm of elongated spermatids (E), although with less intense staining than in the prior stages. Sertoli cells (arrowhead) present LAP1 staining in the NE. (**j**) Expression of several LAP1 isoforms in mouse testis obtained by WB analysis. Essentially, two major bands are detected as previously described in [36]. One of the bands is below 75 kDa and presumably corresponds to both LAP1A and LAP1B, and the other one is above 50 kDa and corresponds to LAP1C; molecular masses in kDa are indicated on the left. LAP1: lamina-associated polypeptide 1; NE: nuclear envelope; WB: Western blot.

Seminiferous tubules representative of all stages of spermatogenesis in mouse were examined by immunohistochemistry (IHC), and differential LAP1 localization in the diverse stages as well as distinct cell types was observed (Figure 1). The negative control did not show cellular LAP1 staining (Figure 1a), validating the following results. First of all, a distinct LAP1 expression is found in the NE of somatic cells (Figure 1b), specifically in peritubular (Figure 1g), Sertoli and Leydig (Figure 1h) cells. Regarding germ cells, LAP1 staining is barely detected in the NE before meiotic cell divisions. Contrastingly, in stages VI–VII (Figure 1c), LAP1 starts to concentrate in one half of the NE, being less prominent in the cytoplasm of round spermatids. This staining pattern becomes progressively more evident from stage VIII (Figure 1d) onwards, which is consistent with the beginning of cellular elongation. In early stage IX (Figure 1e), the previously mentioned signs of LAP1 polarization at one half of the NE are more accentuated. Later, in stage IX (Figure 1f), this polarization coincides with nuclear reshaping and the shifting in directionality of round spermatids to the center of the seminiferous tubule. Throughout this stage, the cytoplasm of elongating spermatids also extends into the tubular lumen as spermiogenesis progresses, which occurs concomitantly with an increase in cytoplasmic LAP1 staining in stage IX elongating spermatids (Figure 1e–f). During stage X (Figure 1g), when dramatic nuclear reshaping into a needle-like structure begins, leading to progressive narrowing of the nucleus, LAP1 is still highly distributed in one half of the NE, and its cytoplasmic localization is also sustained. Finally, in stage XI, the spermatid head forms a distinct protrusion with a sharp angle, and its nucleus becomes thinner, more elongated and stains more intensely, indicating chromatin condensation. During this stage (Figure 1h), LAP1 distributes homogeneously in the cytoplasm of elongating spermatids, and although being circumscribed to one half of the NE, its perinuclear presence decreases drastically. In stage XII (Figure 1i), wherein spermatid elongation continues, LAP1 staining patterns that are observed in previous stages become somewhat diffuse both in the NE and cytoplasm. Overall, in mouse spermatogenesis, LAP1 presents a peculiar subcellular distribution in elongating spermatids from stage VIII onwards (Figure 1b–i). On the one hand, LAP1 is located in one half of the NE, concentrating in the centriolar pole of the spermatid nuclei as nuclear reshaping ensues, which is associated with the development of the manchette. Concomitantly, LAP1 staining appears homogeneously distributed in the elongating cytoplasm, before it becomes progressively poorly detectable in fully-differentiated mature spermatids (stage VI–VII; Figure 1c).

Sodium dodecyl sulfate polyacrylamide gel electrophoresis (SDS-PAGE) followed by immunoblotting analysis was performed to further characterize the presence of LAP1 in mouse testis. The results (Figure 1j) show the expression of multiple immunoreactive bands corresponding to mouse LAP1 isoforms previously described for several mouse cell lines and tissues [36,37,38]. One of the bands is below 75 kDa and presumably corresponds to both LAP1A and LAP1B, and the other band is above 50 kDa and corresponds to LAP1C. Interestingly, various lower intensity bands with molecular weights inferior to 50 kDa are also observed.

**Figure 2:**
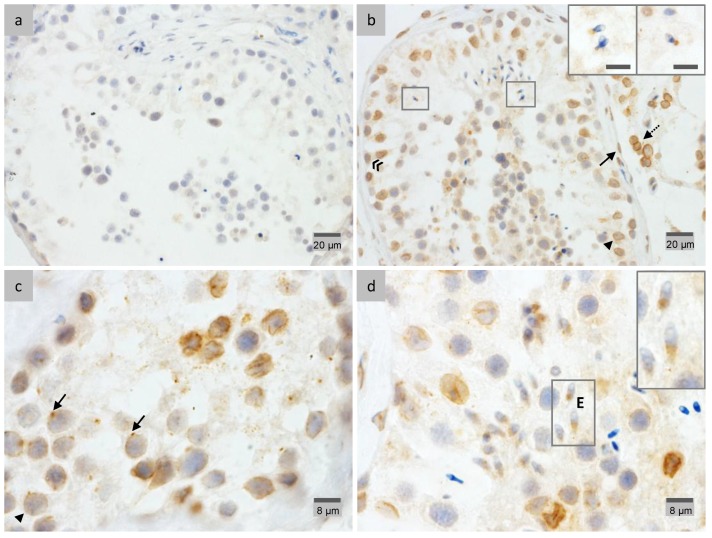
Immunohistochemical localization of LAP1 in human testis sections fixed in 4% PFA. Positive immunostaining appears in brown color and nucleus counterstaining with hematoxylin. (**a**) Negative controls with rabbit IgG showed no LAP1 staining. (**b**) LAP1 is present in the NE of peritubular (arrow), Leydig (dotted arrow) and Sertoli (arrowhead) cells, as well as spermatogonia (double arrowhead). Additionally, LAP1 is found in mature spermatids at the centriolar pole (ROIs, scale bar: 8 μm). (**c**) LAP1 staining is also evident in the NE of spermatocytes and in a dot juxtaposed with the nucleus (arrows). (**d**) LAP1 polarizes at the centriolar pole of elongating spermatids (E), which is concomitant with nuclear reshaping and chromatin condensation. LAP1: lamina-associated polypeptide 1; PFA: paraformaldehyde; NE: nuclear envelope; ROI: region of interest.

IHC analyses for LAP1 distribution throughout mouse spermatogenesis were performed in testis fixed in diluted Bouin’s solution (Figure 1). Despite the fact that this fixation method leads to better conserved tissue morphology [39], fixation with 4% paraformaldehyde (PFA) delivered enhanced staining in the case of LAP1 analysis in human testis (Figure 2). Negative controls (Figure 2a) showed no staining when compared to tissue sections incubated with the specific LAP1 antibody. Overall, LAP1 presence was observed in the NE of somatic cells, namely peritubular, Leydig and Sertoli cells (Figure 2b). Additionally, germ cell distribution of LAP1 in the NE is restricted to spermatogonia and spermatocytes (Figure 2b,c, respectively), which is different from that observed in mouse sections wherein LAP1 staining is found in one half of the NE during the spermatid elongation process. In addition, LAP1 also presents a dotted feature in the cytoplasm of spermatocytes, juxtaposed with the nucleus (Figure 2c), which is unexpected for this membrane-integrating protein. Nevertheless, with the progression of the differentiation process, LAP1 appears exclusively distributed in the centriolar pole of elongating spermatids (Figure 2d) until the formation of elongated mature spermatids, wherein chromatin condensation is clearly evident given the darker staining with hematoxylin (Figure 2b). Assessment of LAP1 cellular and subcellular distribution in human spermatids allowed us to conclude that it has an expression pattern quite similar to that found for mouse spermatids. This INM protein rests in the NE of somatic and pre-meiotic germ cells, but its localization shifts with the beginning of spermiogenesis. Indeed, during spermatid elongation (Figure 2d), LAP1 concentrates at the centriolar pole lasting up until the formation of fully-mature spermatids (Figure 2b), after nuclear reshaping and chromatin condensation.

### 2.2. LAP1 as a New Player in Manchette Development

**Figure 3:**
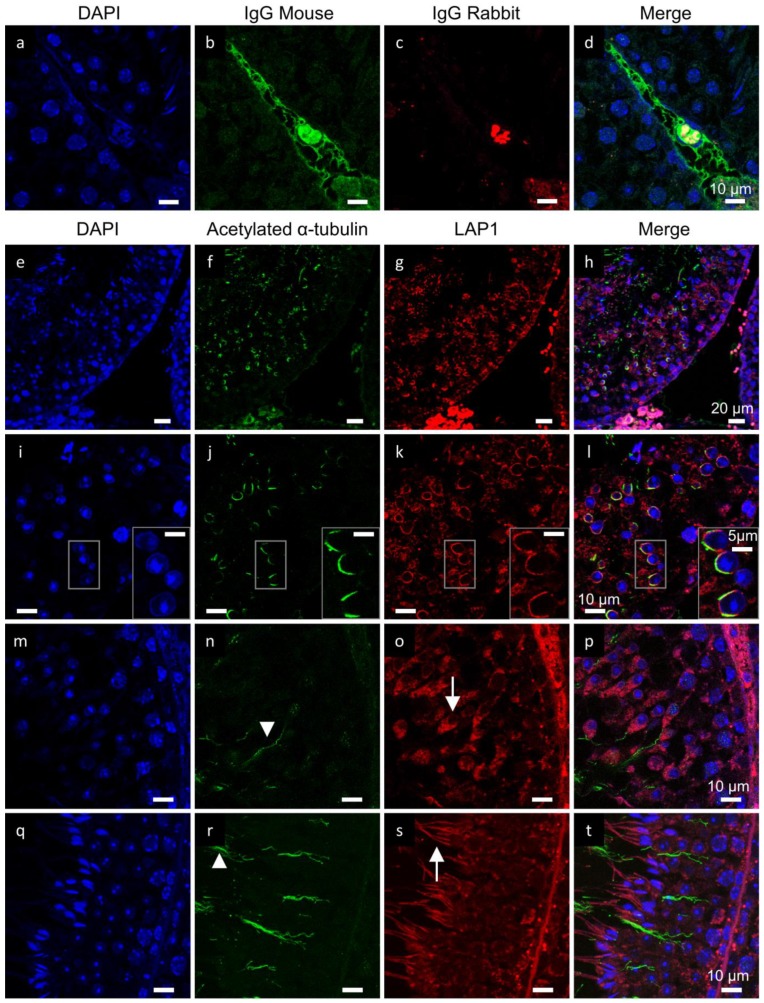
Double immunofluorescence of LAP1 and acetylated α-tubulin in mouse testis sections fixed in diluted Bouin’s solution. DAPI appears in blue, IgG mouse and acetylated α-tubulin in green, IgG rabbit and LAP1 in red. (**a**–**d**) Negative controls with mouse and rabbit IgGs showed some staining in the interstitial tissue through stages VIII–IX. (**e**–**l**) LAP1 polarizes at the NE with acetylated α-tubulin (ROI) at the beginning of spermatid elongation, during stages VIII–IX. (**m**–**p**) As elongation proceeds in stages IX–X, LAP1 appears homogeneously distributed in the cytoplasm of elongating spermatids ((**o**), arrow), while acetylated α-tubulin relocates to the axoneme ((**n**), arrowhead). (**q**–**t**) Upon spermatid maturation in stages VII–VIII, LAP1 concentrates along the midpiece of already elongated spermatids ((**s**), arrow), and acetylated α-tubulin remains in the flagellar axoneme ((**r**), arrowhead). LAP1: lamina-associated polypeptide 1; NE: nuclear envelope; ROI: region of interest.

LAP1 localization was profiled during spermiogenesis, in particular upon manchette formation. Double immunofluorescence staining for LAP1 and acetylated α-tubulin, a characteristic structural element of the manchette in spermatids [40,41], was performed in mouse testis (Figure 3e–t). As the negative control showed no specific staining inside the seminiferous tubules in either of the IgG conditions (Figure 3a–d), it is reasonable to assume that the staining present in the following IHC analyses is specific (Figure 3e–t). Overall examination of mouse seminiferous tubules revealed varying LAP1 staining throughout spermiogenesis, which is consistent with the IHC results previously presented (Figure 1). Once the manchette develops (Figure 3f,j), LAP1 aligns with acetylated α-tubulin in the NE (Figure 3g,h,k,l), coinciding with the beginning of spermatid elongation. By inspecting this region in detail, it is visible that, regardless of sharing exceedingly similar localization patterns, these proteins do not colocalize perfectly (Figure 3l, ROI). In fact, it seems that LAP1 is concentrated in the INM of the NE, surrounded externally by acetylated α-tubulin on the cytosolic side. Further along the elongation process (Figure 3m–p), LAP1 appears homogeneously distributed in the cytoplasm between the nucleus and the axoneme as spermiogenesis proceeds, while acetylated α-tubulin relocates to the axoneme. Upon spermatid maturation (Figure 3q–t), LAP1 concentrates along the midpiece of already elongated spermatids, while acetylated α-tubulin remains located in the flagellar axoneme.

### 2.3. PP1γ2 Shares Similar Subcellular Distribution with LAP1 in the Testis

**Figure 4:**
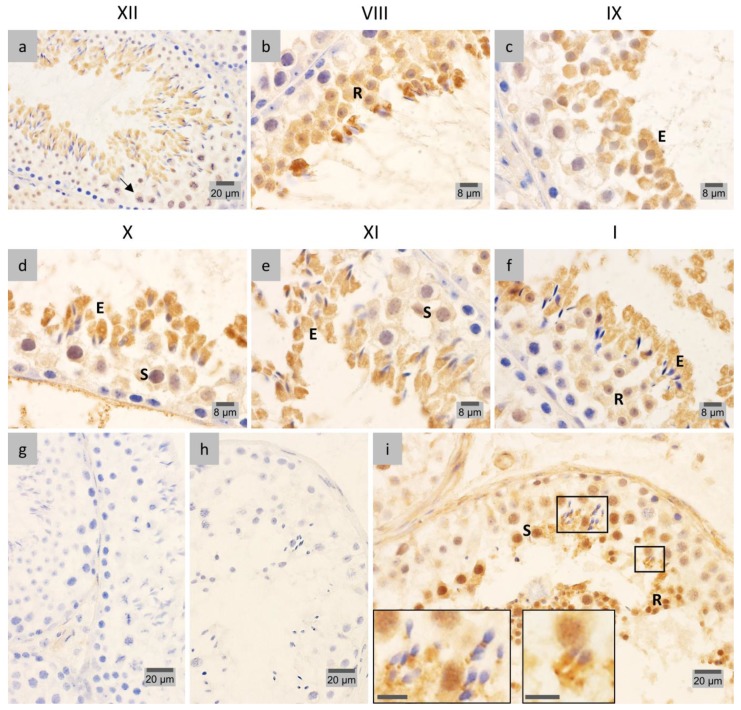
Immunohistochemical localization of PP1γ2 in mouse (**a**–**f**) and human (**g**–**i**) testis sections fixed in diluted Bouin’s solution. Positive immunostaining appears in brown color and nucleus counterstaining with hematoxylin. (**a**–**f**) Mouse spermatogenic stages wherein PP1γ2 expression is concomitant with LAP1 are depicted. (**a**) In stage XII seminiferous tubules (stages are indicated with Roman numerals), PP1γ2 concentrates in the cytoplasm of meiotic cells (arrow) and elongating spermatids. (**b**) Stage VIII round spermatids (R) present staining for PP1γ2 in both the nucleus and cytoplasm, whereas a high staining is visible in residual bodies of elongated mature spermatids. (**c**) High positive staining for PP1γ2 in the cytoplasm, centriolar pole and one half of the nucleus in stage IX elongating spermatids (E). (**d**) In stage X, in addition to PP1γ2 being found in the cytoplasm and nucleus of elongating spermatids (E), the same subcellular distribution is observed in spermatocytes (S). (**e**) Prior to meiosis, PP1γ2 distinctively stains the nucleus and cytoplasm of spermatocytes (S), but only the cytoplasm of stage XI elongating spermatids (E). (**f**) In stage I, round spermatids (R) show equal PP1γ2 staining in the nucleus and cytoplasm, while elongated spermatids (E) remain stained only in the cytoplasm. (**g**–**i**) PP1γ2 expression in human spermatogenic cells is depicted. (**i**) PP1γ2 concentrates in the nucleus and cytoplasm of pachytene spermatocytes (S), as well as round spermatids (R), progressively shifting to the centriolar pole of elongating spermatids (right ROI, scale bar: 8 μm) and elongated mature spermatids (left ROI, scale bar: 8 μm). (**g**,**h**) Negative controls showed no PP1γ2 staining. PP1: protein phosphatase 1. ROI: region of interest.

The cellular and subcellular localization of PP1γ2, the testis-enriched and sperm-specific PP1 isoform, was investigated during the different stages of spermatogenesis in both mouse (Figure 4a–f) and human (Figure 4g–i) testicular cells. In mouse, IHC analysis of PP1γ2 distribution throughout spermiogenesis revealed a stage-specific pattern that resembles the one found for LAP1. Accordingly, the PP1γ2 signal is quite noticeable in the nucleus and cytoplasm of stage VIII round spermatids (Figure 4b), as is the case of LAP1. As cellular elongation proceeds in stage IX, PP1γ2 is distributed in the centriolar pole, in one half of the nucleus, and widely in the cytoplasm (Figure 4c), concomitant with LAP1 staining. Later in stage X, spermatids present nuclear reshaping and cytoplasm elongation, with PP1γ2 immunostaining being found in the nucleus and cytoplasm (Figure 4d), again similar to LAP1 localization. After complete narrowing of the nucleus into a needle-like shape, PP1γ2 is located only in the extended cytoplasm of stage XI (Figure 4e) and stage XII (Figure 4a) spermatids, a distribution that is preserved in fully-elongated spermatids in stage I (Figure 4f), identical to the case of LAP1. Therefore, the cellular and subcellular localization patterns of PP1γ2 and LAP1 show extraordinary similarities during the various spermiogenic stages. Overall, throughout the cycle of spermatogenesis in mouse testis, PP1γ2 is present in the nucleus and cytoplasm of spermatocytes before meiosis (Figure 4d–e), but then distributes all over the cytoplasm in meiotic germ cells (Figure 4a), after which it returns to be located in both the nucleus and cytoplasm of round spermatids (Figure 4b,f). Lastly and most prominently, PP1γ2 becomes dispersed in the cytoplasm of elongating spermatids (Figure 4a,e), until excision of residual bodies of elongated mature spermatids, where PP1γ2 is concentrated at the end of spermiogenesis (Figure 4b). Despite being thoroughly described in mouse testis, the differential localization of PP1 isoforms in human spermatogenesis has not been investigated by IHC. In this study, IHC analysis of PP1γ2 cellular and subcellular distribution in human testicular cells (Figure 4i) revealed that this phosphatase concentrates in the nucleus and cytoplasm of pachytene spermatocytes prior to meiosis. Later on, during spermiogenesis, PP1γ2 staining shifts from the nucleus and cytoplasm of round spermatids to the centriolar pole of elongating spermatids (Figure 4i, right ROI) and completely elongated mature spermatids (Figure 4i, left ROI). The negative controls (Figure 4g,h) in mouse and human tissue sections, respectively, reveal no unspecific PP1γ2 staining; hence, the corresponding results (Figure 4) are considered specific.

### 2.4. LAP1 Chromatin Regulation through Lamin Interaction

**Figure 5:**
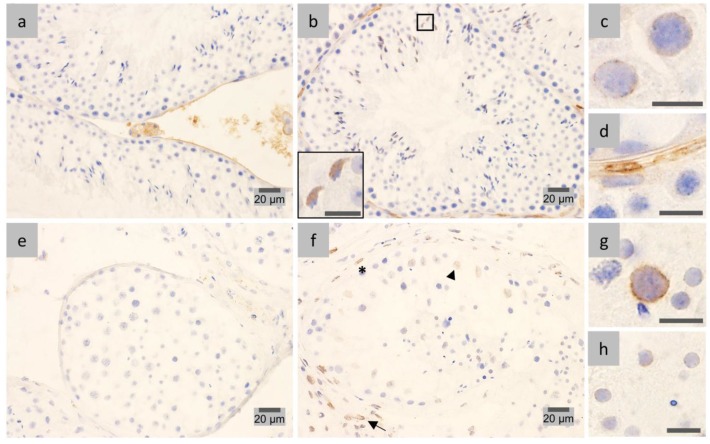
Immunohistochemical localization of lamin A/C in mouse (**a**–**d**) and human (**e**–**h**) testis sections fixed in diluted Bouin’s solution. Positive immunostaining appears in brown color and nucleus counterstaining with hematoxylin. (**a**,**e**) Negative controls revealed weak lamin A/C staining in the cytoplasm of interstitial tissue. (**b**) Mouse seminiferous tubule stained for lamin A/C shows localization most evident in the acrosome of elongated mature spermatids (ROI, scale bar: 8 μm), as well as (**c**) in the NE of pachytene spermatocytes and (**d**) peritubular cells (scale bars: 8 μm). (**f**) In human testis, lamin A/C staining is observed in the NE of peritubular (asterisk), Leydig (arrow) and Sertoli (arrowhead) cells, as well as (**g**) pachytene spermatocytes, (**h**) with a particular weak signal being found in round spermatids (scale bars: 8 μm). NE: nuclear envelope; ROI: region of interest.

Although the presence and distribution of nuclear lamins in mouse and human testes has been previously investigated, it is not fully clarified. Overall, observations of lamin A/C staining in mouse seminiferous tubules identified no positive signal in spermatogonia, Sertoli cells or round spermatids (Figure 5b). In contrast, lamin A/C is present in the NE of pachytene spermatocytes (Figure 5c) and peritubular cells (Figure 5d). Ultimately, lamin A/C is most prominently expressed in the acrosome of elongated mature spermatids (Figure 5b). The negative IgG control (Figure 5a) revealed some unspecific staining in Leydig cells; thus, it is not possible to assess lamin A/C staining in these somatic cells. In human seminiferous tubules, lamin A/C is located in the NE of peritubular, Leydig and Sertoli cells (Figure 5f), as well as pachytene spermatocytes (Figure 5g). Of note, we report for the first time a particular weak staining in the NE of round spermatids (Figure 5h). The negative controls (Figure 5a,e) did not show unspecific lamin A/C staining, so that the corresponding results (Figure 5) are deemed specific.

**Figure 6:**
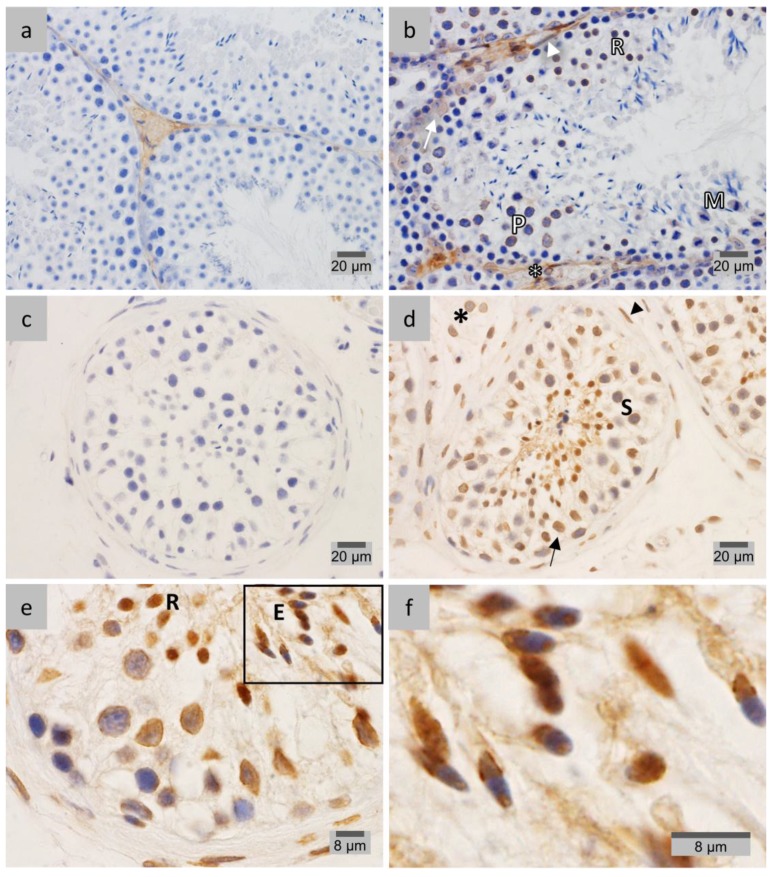
Immunohistochemical localization of lamin B1 in mouse testis sections fixed in diluted Bouin’s solution (**a**,**b**) and human testis sections fixed in 4% PFA (**c**–**f**). Positive immunostaining appears in brown color and nucleus counterstaining with hematoxylin. (**a**,**c**) Negative controls showed lamin B1 staining in the interstitial tissue. (**b**) In mouse testis, lamin B1 is present in the NE of peritubular (arrowhead), Leydig (asterisk) and Sertoli (arrow) cells, spermatogonia, pachytene spermatocytes (P) and round spermatids (R). Lamin B1 is also distributed in the cytoplasm of meiotic cells (M). (**d**) In human seminiferous tubules, lamin B1 is clearly detected in the NE of peritubular (arrowhead), Leydig (asterisk) and Sertoli (arrow) cells, in addition to spermatocytes (S). (**e**) In spermiogenic cells, lamin B1 is found in the nucleus of round spermatids (R). Additionally, lamin B1 is located in the centriolar pole of elongating and elongated mature spermatids (E). (**f**) ROI from Figure 6e. PFA: paraformaldehyde; NE: nuclear envelope; ROI: region of interest.

As regards lamin B1 cellular and subcellular localization during mouse spermatogenesis, a positive signal is generally detected in the NE of peritubular, Leydig and Sertoli cells, in addition to different pre- and post-meiotic germ cells, namely spermatogonia, pachytene spermatocytes and round spermatids (Figure 6b). Interestingly, lamin B1 is dispersed throughout the cytoplasm of meiotic cells (Figure 6b), whereas no staining was observed in spermiogenic cells following round spermatids. In human testis (Figure 6d–f), lamin B1 is clearly expressed in the NE of peritubular, Leydig and Sertoli cells, as well as spermatocytes (Figure 6d). Additionally, lamin B1 staining is present in the nucleus of round spermatids (Figure 6e) and in the centriolar pole of both elongating and elongated mature spermatids (Figure 6f). The negative controls (Figure 6a,c) revealed no unspecific lamin B1 staining; hence, the corresponding results (Figure 6) are considered specific. These findings are consistent with previous reports [26,33].

### 2.5. γ-Tubulin Localizes in the Centriolar Pole during Spermiogenesis

**Figure 7:**
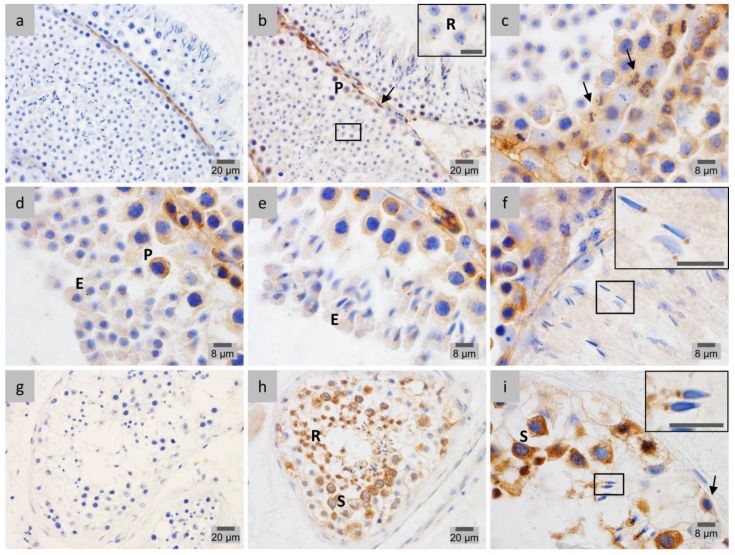
Immunohistochemical localization of γ-tubulin in mouse testis sections fixed in diluted Bouin’s solution (**a**–**f**) and human testis sections fixed in 4% PFA (**g**–**i**). Positive immunostaining appears in brown color and nucleus counterstaining with hematoxylin. (**a**,**g**) Negative controls showed γ-tubulin staining in the interstitial tissue. (**b**) In mouse seminiferous tubules, γ-tubulin is present in the cytoplasm of pre-meiotic germ cells, namely spermatogonia (arrow) and pachytene spermatocytes (P). From meiosis onwards, γ-tubulin concentrates in dots at the centriolar poles, starting in round spermatids (R) (ROI, scale bar: 8 μm). (**c**) Typically, spermatogonia undergoing mitosis also express γ-tubulin in centrosomes (arrows). (**d**) γ-Tubulin concentrates in the cytoplasm of pachytene spermatocytes (P). (**d**,**e**) As spermatid elongation proceeds, the dot at the centriolar pole remains in elongating spermatids (E). (**f**) Centriolar staining for γ-tubulin is observed until maturation of elongated spermatids (ROI, scale bar: 8 μm). (**h**) In human testis, after meiosis, γ-tubulin accumulates in dots at the centriolar poles and is also distributed homogenously across the cytoplasm of round spermatids (R). (**i**) In pre-meiotic germ cells, from spermatogonia (arrow) to spermatocytes (S), γ-tubulin staining appears dispersed throughout the cytoplasm. In completely elongated spermatids, γ-tubulin (ROI, scale bar: 8 μm) still concentrates in a dot at the centriolar pole. PFA: paraformaldehyde. ROI: region of interest.

The presence of γ-tubulin in rodents has been previously characterized and reported to be most prominent in spermatids as a marker of the centriolar pole [35,42,43,44]. In this study, the cellular and subcellular distribution of γ-tubulin in both mouse and human testes was analyzed and compared to the corresponding pattern obtained for LAP1. In mouse testicular tissue, γ-tubulin stains the cytoplasm of germ cells from initial phases of the spermatogenic cycle, including spermatogonia (Figure 7b) and pachytene spermatocytes (Figure 7b,d). However, from meiosis onwards, γ-tubulin accumulates in dots at the centriolar poles from round spermatids (Figure 7b) to fully-elongated mature spermatids (Figure 7d–f). Additionally, in mitotic spermatogonia, two dots are observed at the centriolar regions (Figure 7c). Throughout the human cycle of spermatogenesis, γ-tubulin staining is quite similar to that described in mouse seminiferous tubules. In pre-meiotic germ cells, ranging from spermatogonia to spermatocytes, γ-tubulin appears dispersed all over the cytoplasm (Figure 7i). Following meiosis, γ-tubulin is concentrated in dots at the centriolar poles, while being also distributed homogenously in the cytoplasm of round spermatids (Figure 7h). In completely elongated mature spermatids, γ-tubulin concentrates in a dot at the centriolar pole (Figure 7i). Other than peritubular cells, no γ-tubulin signal was detected in the negative controls for mouse and human tissue sections (Figure 7a,g, respectively); therefore, the corresponding γ-tubulin staining results (Figure 7) are considered specific.

## 3. Discussion

For the first time, we describe the cellular and subcellular distribution pattern of LAP1 in the seminiferous epithelium during the differentiation of germ cells into highly specialized sperm cells in both mouse and human testes. The present work referring to mouse spermatogenesis demonstrates a prominent LAP1 expression in the NE of round spermatids just before the initiation of spermatid elongation. This expression pattern shifts to the centriolar pole during elongation, before LAP1 perinuclear staining becomes poorly detectable in fully-mature spermatids, which are then released from the seminiferous epithelium into the tubular lumen. Despite being previously associated with cell cycle progression, NE integrity, DYT1 dystonia and skeletal muscle maintenance [14,15,16,45], LAP1 functional characterization is still not fully elucidated. Interestingly, it was not possible to verify the expected presence of LAP1 during meiosis, and rather surprisingly, LAP1 staining was mainly detected throughout mouse spermiogenesis (Figure 1 and Table 1). The localization of LAP1 in the NE of somatic cells, including peritubular, Sertoli and Leydig cells, seems to be in accordance with the previously described roles of LAP1 in nuclear architecture maintenance [14]. In stage VIII, when round spermatids begin the elongation process, LAP1 is highly expressed and, most importantly, relocates to the centriolar pole of the NE. In addition, LAP1 appears dispersed in the cytoplasm of round spermatids, which is quite unusual considering the typical localization of this INM protein in the NE. In stage IX, nuclear reshaping and simultaneous redirection of round spermatids towards the center of the seminiferous tubule lumen occur concomitantly with increasing LAP1 expression in both the NE centriolar pole and cytoplasm. In stage XI, as chromatin compaction ceases, LAP1 is still circumscribed to one half of the NE, but its presence decreases drastically in comparison to the previous stages. The distribution of LAP1 is partially analogous to that observed for other NE proteins, namely LBR, LAP2, SUN domain-containing protein 1 (SUN1) and 3 (SUN3), lamins B1 and B3. In round and elongating spermatids, these proteins also shift to the NE centriolar pole, but they remain only in the nuclear periphery [6,11,12,32]. As in the case of LAP1, the significance of the redistribution of these NE proteins is presently unknown. However, alongside LAP1, NE proteins might be involved in chromatin reorganization associated with spermiogenesis. Previous reports extensively described that NE proteins, in particular lamins and its interactors, play an important role in chromatin dynamics and function (reviewed in [46]). By moving to the posterior pole, chromatin binding sites at the NE would contribute to the achievement of non-random, sperm-specific chromatin distribution. In fact, LAP1 becomes progressively poorly detectable in the NE centriolar pole by the end of the spermatid elongation process. According to the results herein reported, we propose that the progressive disappearance of perinuclear LAP1 observed in fully-elongated mature spermatids may be a consequence of the ending of the chromatin condensation process. From stage IX–XI, the homogenous signal of cytoplasmic LAP1 also increases exponentially in elongating spermatids. The significance of this quite unusual distribution of LAP1 is currently unknown. However, the widespread cytoplasmic presence of LAP1 might be translated from its distribution in mitosis, wherein LAP1 is dispersed throughout the cytoplasm in a punctuate pattern, colocalizing with α-tubulin in the mitotic spindle and with γ-tubulin in centrosomes [15]. Importantly, not only is LAP1 crucial for centrosome positioning near the NE, but also its depletion results in a decrease of mitotic cells and in the levels of acetylated α-tubulin and lamin B1 [15]. This functional association might be reproduced during spermiogenesis, as dynamic microtubules (MTs) are essential for the assembly of various MT-based structures that participate in spermatid remodeling, such as manchette formation and nuclear reshaping [47]. In fact, the data presented here show that LAP1 accompanies manchette formation, since it colocalizes with α-acetylated tubulin during mouse spermiogenesis (Figure 3). Furthermore, LAP1 expression in the NE of elongating spermatids, concentrating in their centriolar pole, correlates exactly with manchette development. Taking these facts into account, it will be of interest to understand how LAP1 might be regulating or communicating with these cytoskeletal elements and how it is connected to the elongating spermatid nucleus. Therefore, our results not only suggest coherence between manchette formation and relocation of LAP1 to the NE centriolar pole, but also provide the first evidence for a putative involvement of LAP1 in linking nuclear lamina to cytoskeletal elements.

Additionally, the data herein presented disclose the expression of LAP1 in mouse testis by SDS-PAGE and immunoblotting analysis (Figure 1j). Interestingly, it was possible to observe the presence of numerous LAP1 immunoreactive bands. In light of a recent study by Kuhn et al., it is conceivable that these represent fragments originated from proteolytic cleavage of the LAP1 N-terminal membrane anchor by signal peptide peptidase like 3 (SPPL3) [48]. In fact, this is most likely the case for all of the multiple lower intensity bands with molecular weights under 50 kDa, but this should be further investigated. The presence of LAP1 in human testis, by SDS-PAGE and immunoblotting analyses, was previously characterized by our group [19].

In the present study, it was also demonstrated that LAP1 distribution in the human spermatogenic cycle shares various similarities with that of mouse spermatogenesis (Figure 1 and Figure 2; Table 1 and Table 2). This INM membrane protein rests in the NE of germ cells until meiotic division, but its localization shifts throughout spermiogenesis. During spermatid elongation, LAP1 concentrates at the centriolar pole, reinforcing the hypothesis that LAP1 might function as a cytoskeleton regulator of spermiogenesis and that simultaneously it might be coordinating chromatin tethering and condensation. Interestingly, there is a particular difference between the localization profile of LAP1 in mouse and human testes. LAP1 presents a dotted distribution in the cytoplasm of human spermatocytes, which is quite novel for this membrane integrating protein. The reasons behind this specific localization are still unknown, but one might speculate that it is related with centrosome positioning.

The distribution patterns of some LAP1-interacting proteins were also identified in both mouse and human spermatogenesis, adding novel insights to their cellular and subcellular localization throughout the spermatogenic cycle. During mouse and human spermiogenesis, PP1γ2 staining shares an extraordinary similar pattern to that of LAP1, due to their homogenous distribution in the cytoplasm at the posterior pole (Figure 4; Table 1 and Table 2). These results complement and confirm previous reports for PP1γ2 distribution in mouse [23], but reveal for the first time the expression pattern of this protein in human spermatogenesis. When exploring the putative interaction between LAP1 and PP1γ2 in spermiogenesis, functional perspectives arise, as LAP1 has been previously identified to be a PP1γ substrate [18,19]. Given that both proteins have important roles in mitosis regarding MT dynamics [15,49], there is an additional putative significance of this association in the spermatid elongation process.

There is increasing evidence that the nuclear lamina plays a crucial role in spermatid differentiation during spermiogenesis. With this work, it was possible to add to and confirm previous reports for lamin A/C distribution in mouse testis (Figure 5; Table 1) [28,29]. In fact, lamin A/C is most prominently expressed in the NE of pachytene spermatocytes and the acrosome of elongated mature spermatids. In human testis (Figure 5; Table 2), this lamin isoform seems to be present in the NE of pachytene spermatocytes and, rather faintly, of round spermatids, adding new findings to previous studies [26,33]. Given the distribution pattern of lamin A/C, LAP1 does not seem to share the same expression profile throughout spermatogenesis either in mouse or human testes. Concerning lamin B1, the present results supplement the available literature for B-type lamins [27,28], finally defining lamin B1 distribution throughout mouse spermatogenesis (Figure 6; Table 1) and its presence in the NE of peritubular, Leydig and Sertoli cells. In pre-meiotic stages, lamin B1 localizes to the NE of spermatogonia and pachytene spermatocytes, then it is dispersed in the cytoplasm of meiotic cells and finally returns to the NE of round spermatids. In human testis (Figure 6; Table 2), our results first demonstrate the presence of lamin B1 in the NE of spermatocytes and also confirm the results from previous studies [26], wherein lamin B1 is clearly expressed in the nucleus of round spermatids. As elongation ensues, lamin B1 concentrates in the posterior pole until the formation of fully-elongated mature spermatids. Taken together, the present results indicate a correlation between the presence of nuclear lamina and an increase in the morphologic flexibility of the nucleus, enabling intense nuclear remodeling during spermiogenesis, either in the acrosome cap (lamin A/C) or at the centriolar pole (lamin B1). Additionally, the presence of lamin B1 at the centriolar pole is quite similar to LAP1 distribution in the NE, both in mouse and human spermiogenesis. Once again, these results reinforce the putative role of LAP1 and lamins in the regulation of chromatin condensation and simultaneous nuclear reshaping during the process of spermiogenesis.

The presence of γ-tubulin in rodent testis tissue has been previously characterized, being most prominent in spermatids [35,42,43,44]. The work herein described shows that, throughout spermatogenesis, γ-tubulin distribution is similar in mouse and human seminiferous tubules (Figure 7; Table 1 and Table 2). Interestingly, our results show that, in the initial phases of the spermatogenic cycle, both spermatogonia and spermatocytes express homogeneously γ-tubulin in the cytoplasm. However, from meiosis onwards, until spermatid maturation, γ-tubulin concentrates in dots at the centriolar poles of spermiogenic cells. The comparative study of the expression profiles of LAP1 and γ-tubulin in mouse and human spermatogenesis reveals an interesting association. The cytoplasmic distribution of γ-tubulin in round and elongating spermatids coincides with LAP1 localization in the centriolar pole. Additionally, in fully-elongated mature spermatids, the dotted pattern at the centriolar pole is observed in both LAP1 and γ-tubulin staining in human testis (Figure 2b and Figure 7i, respectively). Further analysis of this association might clarify and reveal interesting functions for LAP1 regarding MT-based structures and their organization. In fact, after the initiation of axoneme formation, flagella development continues during the elongation phase of spermiogenesis. Thus, the cytoplasmic presence of LAP1 in elongating spermatids might be related to the recently described association of LAP1 to the centrosome-cilium network [50] and should be further investigated.

## 4. Materials and Methods

### 4.1. Antibodies

Several primary antibodies were used to detect multiple proteins analyzed in the experimental procedures, namely anti-LAP1, anti-lamin A/C, anti-lamin B1, anti-PP1γ2, anti-acetylated α-tubulin and anti-γ-tubulin (Table 3). Additionally, as a negative control for IHC, isotype mouse and rabbit IgGs (Vector Laboratories, Burlingame, CA, USA) were used instead of the primary antibody in the corresponding concentrations. Specific secondary antibodies were used in accordance with the particular technique performed, as described below. To visualize immunohistochemical preparations with the colorimetric method, testis tissue sections were incubated with Powervision Poly-HRP anti-Ms/Rb/Rt IgG (Leica Biosystems, Nussloch, Germany) or Powervision Poly-HRP anti-Ms IgG (Leica Biosystems, Nussloch, Germany). In immunofluorescent colocalization assays, tissue sections were incubated with Alexa Fluor 594 (goat anti-rabbit IgG; Invitrogen, Thermo Fisher Scientific, Waltham, MA, USA) and Alexa Fluor 488 (goat anti-mouse IgG; Invitrogen, Thermo Fisher Scientific, Waltham, MA, USA), both diluted 1:300. For immunoblotting, the IRDye 800CW anti-rabbit secondary antibody was used, diluted 1:5000 (LI-COR Biosciences, Lincoln, NE, USA).

### 4.2. Animals

Adult male Balb/c mice (8 weeks old) testes were used for IHC analyses. The animals were used and maintained according to regulations provided by the animal ethical committee of the Academic Medical Center (Amsterdam, The Netherlands), which also approved the experiments. For Western blot (WB) analyses, male adult C57BL/6 mice (8 weeks old) testis samples were obtained from the University of Minho (Braga, Portugal). All experiments were conducted in accordance with the Portuguese national authority for animal experimentation, Direcção Geral de Veterinária (ID: DGV9458), which approved the experiment. Animals were kept in accordance with the guidelines for the care and handling of laboratory animals in the Directive 2010/63/EU of the European Parliament and of the Council [51].

### 4.3. Patient Material

For IHC, testicular material was donated after informed consent by four patients undergoing bilateral orchidectomy as part of prostate cancer treatment (Patients URO0179, URO0368, URO0059 and URO0126). According to Dutch law, approval of the ethics committee was not required because anonymized tissue samples were used. None of the patients had previously received chemotherapy or radiotherapy, and the morphology of the testes showed normal spermatogenesis in all cases. Testis biopsies were fixed in 4% paraformaldehyde (PFA) or diluted Bouin’s solution and embedded in paraffin. All of the biopsies and following procedures were performed according to regulations from the Academic Medical Center (Amsterdam, The Netherlands).

### 4.4. Tissue Preparation for IHC

Tissue samples from both human and mouse were previously fixed in diluted Bouin’s solution or 4% PFA and paraffin embedded. The two fixatives were used in order to optimize the staining process for each of the primary antibodies used in IHC. The sections were cut in the microtome (thickness of 5 μm per slice) and placed in a water bath (37 °C). Tissue sections were set into adhesive microscope slides (KP-Silan adhesive slides grounded-90; Klinipath BV). Slides were then placed on the heating plate at 42 °C to stretch the tissue homogeneously and incubated overnight at 37 °C to dry the tissue completely.

### 4.5. Histological Staining

Tissue sections were deparaffinized, and antigen retrieval was performed in 10 mM sodium citrate, pH 6.0, with microwave oven irradiation for 2 min at maximum Watt, followed by heating for 10 min at minimum Watt, without boiling. As this procedure depends on the specific protein that is being analyzed, all of the primary antibodies were tested in three conditions: no antigen retrieval, one-time antigen retrieval or three-time antigen retrieval. Endogenous peroxidase was blocked with 0.3% H_2_O_2_/PBS. Nonspecific adhesion sites were blocked for 1 h at room temperature, in a humidified chamber. The blocking solutions for each antibody were optimized (Table 3). Sections were subsequently incubated with the primary antibodies (Table 3) or with IgGs as negative controls, for 2 h at room temperature.

To visualize immunohistochemical preparations with the colorimetric method, tissue sections were incubated with the respective Poly-HRP secondary antibodies, and the signal was developed with Bright-DAB (Immunologic, Duiven, The Netherlands). Finally, tissue sections were counterstained with freshly filtrated hematoxylin, dehydrated and mounted in Entellan. Slides were examined and photographs were taken using an Olympus BX41 bright-field microscope with an Olympus DP20 color camera (Olympus, Shinjuku, Tokyo, Japan).

In order to detect proteins by immunofluorescence, tissue sections were incubated with the respective fluorescent secondary antibodies. Finally, the preparations were incubated with VECTASHIELD^®^ Mounting Media with DAPI (Vector Laboratories, Burlingame, CA, USA) and visualized by confocal microscopy using the LSM 510-Meta confocal microscope (Zeiss, Oberkochen, Germany) and a 100×/1.4 oil immersion objective. The argon laser lines of 405 nm, 488 nm and a 561-nm DPSS laser were used. Microphotographs were acquired in a sole section in the *Z*-axis (*xy* mode) and represent the mean of 16 scans [52].

### 4.6. Tissue Homogenization for WB Analysis

Mouse testes were removed surgically after the animals were transcardially perfused with 0.9% saline solution. Lysis buffer (50 mM Tris-HCl (pH 8.0), 120 mM NaCl, 4% w/v 3[(3-cholamidopropyl) dimethylammonio]-propanesulfonic acid (CHAPS)) containing a protease inhibitor cocktail were added to tissues, which were then homogenized in a variable speed motor drive homogenizer (750–1000 rpm; Yellowline OST 20 Digital, IKA Labortechnik, Staufen, Germany) with a pestle tissue grinder. Subsequently, homogenized tissues were transferred to a sterile tube and centrifuged at 1000 relative centrifugal force (rcf) and 4 °C for 2 min. The supernatant was collected and sonicated on ice for 10 s (0.5 cycles, 60% amplitude; Sonicator U200S control, IKA Laboratechnik, Staufen, Germany).

### 4.7. SDS-PAGE and Immunoblotting

Total protein concentration of tissue lysates was determined using the BCA Protein Assay kit (Thermo Scientific Pierce, Thermo Fisher Scientific, Waltham, MA, USA). Samples were separated on SDS-PAGE (5%–20% gradient gel) and electrophoretically transferred onto a nitrocellulose membrane (0.2-μm pore size; Whatman^®^). Immunoblotting was performed by blocking the membranes in 5% BSA in Tris-buffered saline containing 0.1% Tween-20 (TBS-T). Membranes were then incubated with anti-LAP1 primary antibody in 3% BSA in TBS-T overnight, at 4 °C (Table 3). After washing in TBS-T, membranes were incubated with IRDye 800CW anti-rabbit secondary antibody in 3% BSA in TBS-T. Detection and image acquisition were achieved with the Odyssey Infrared Imaging system (LI-COR Biosciences, Lincoln, NE, USA).

## 5. Conclusions

Throughout the years, researchers have established that the NE undergoes significant changes in the course of spermiogenesis. Therefore, nuclear reshaping and condensation processes seem to have a highly important role in the development of healthy sperm cells. This study contributes to the identification of LAP1 as a putative functional player during this differentiation process. Initially, by moving to the posterior pole, LAP1 can contribute to the achievement of non-random, sperm-specific chromatin distribution, as well as modulate cellular reshaping features. Furthermore, this NE protein might be associated with the dynamic MT-based morphologic changes associated with manchette formation and flagellar development.

## Figures and Tables

**Table 1:** Cellular and subcellular localization of LAP1 and its interactors during the cycle of spermatogenesis in mouse testis.

Cell Type	Stage/Step	LAP1	PP1γ2	Lamin A/C	Lamin B1	γ-Tubulin
Spermatogonia	–	NE	–	–	NE	Cy
Spermatocytes	*All*	NE	–	–	–	–
	*Pachytene*	–	Nu, Cy	NE	NE	Cy
Round spermatids	*Steps 1–5*	Cy	Nu, Cy	–	NE	CP
	*Steps 6, 7*	NE, Cy	Nu, Cy	–	NE	CP
	*Step 8*	NE, CP, Cy	Nu, Cy	–	NE	CP
Elongating spermatids	*Step 9*	CP, Cy	CP, Nu, Cy	–	–	CP
	*Step 10*	CP, Cy	Nu, Cy	–	–	CP
	*Step 11*	CP, Cy	Cy	–	–	CP
	*Steps 12, 13*	CP, Cy	Cy	–	–	CP
Elongated mature spermatids	*Steps 14, 15*	CP, Cy	Cy	Ac	–	CP
	*Step 16*	–	RB	Ac	–	CP

Steps 1–16: 16 steps of spermiogenesis, including the Golgi (Steps 1–3), capping (Steps 4–7), acrosomal (Steps 8–13) and maturation (Steps 14–16) phases. LAP1: lamina-associated polypeptide 1; PP1: protein phosphatase 1; NE: nuclear envelope (wide distribution); Cy: cytoplasm (wide distribution); Nu: nucleus; CP: centriolar pole; Ac: acrosome; RB: residual body.

**Table 2:** Cellular and subcellular localization of LAP1 and its interactors during the cycle of spermatogenesis in human testis.

Cell Type	Stage	LAP1	PP1γ2	Lamin A/C	Lamin B1	γ-Tubulin
Spermatogonia	–	NE	–	–	–	Cy
Spermatocytes	*All*	NE, Cy_N_	–	–	NE	Cy
	*Pachytene*	–	Nu, Cy	NE	–	–
Round spermatids	–	CP, Cy	Nu, Cy	NE	Nu	CP, Cy
Elongating spermatids	–	CP	CP	–	CP	CP
Elongated mature spermatids	–	CP	CP	–	CP	CP

LAP1: lamina-associated polypeptide 1; PP1: protein phosphatase 1; NE: nuclear envelope (wide distribution); Cy: cytoplasm (wide distribution); Cy_N_: cytoplasm juxtaposed to the nucleus; Nu: nucleus; CP: centriolar pole.

**Table 3:** Primary antibodies used to detect LAP1 and its interactors by immunohistochemical analysis, immunofluorescent colocalization assays and immunoblotting.

Antibody	Origin/Supplier	Antibody Type	Target	Dilution IHC	Blocking Solution
Anti-LAP1	Goodchild and Dauer [45]	Rabbit polyclonal	LAP1	1:2000	5% BSA + 0.01% BSA-C
Anti-lamin A/C	Cell Signaling Technology #2023 (Beverly, MA, USA)	Rabbit polyclonal	Lamin A/C	1:50	3% BSA
Anti-lamin B1 (H-90)	Santa Cruz Biotechnology sc-20682 (Dallas, TX, USA)	Rabbit polyclonal	Lamin B1	1:100	5% BSA
Anti-PP1γ2 (CBC502)	Smith et al. [22]	Rabbit polyclonal	PP1γ2	1:500	3% BSA
Anti-acetylated α-tubulin	Sigma T7451 (St. Louis, MO, USA)	Mouse monoclonal	Acetylated α-tubulin	1:250	5% BSA + 0.01% BSA-C
Anti-γ-tubulin (GTU-88)	Sigma T5326 (St. Louis, MO, USA)	Mouse monoclonal	γ-Tubulin	1:2000	5% BSA + 0.01% BSA-C

LAP1: lamina-associated polypeptide 1; PP1: protein phosphatase 1; BSA: bovine serum albumin; BSA-C: acetylated bovine serum albumin.
